# The Effect of Pollen Source vs. Flower Type on Progeny Performance and Seed Predation under Contrasting Light Environments in a Cleistogamous Herb

**DOI:** 10.1371/journal.pone.0080934

**Published:** 2013-11-15

**Authors:** Miguel A. Munguía-Rosas, María J. Campos-Navarrete, Víctor Parra-Tabla

**Affiliations:** 1 Departamento de Ecología Humana, Centro de Investigación y de Estudios Avanzados del Instituto Politécnico Nacional, Mérida, Yucatán, México; 2 Departamento de Ecología Tropical, Universidad Autónoma de Yucatán, Mérida, Yucatán, México; University of New South Wales, Australia

## Abstract

Dimorphic cleistogamy is a specialized form of mixed mating system where a single plant produces both open, potentially outcrossed chasmogamous (CH) and closed, obligately self-pollinated cleistogamous (CL) flowers. Typically, CH flowers and seeds are bigger and energetically more costly than those of CL. Although the effects of inbreeding and floral dimorphism are critical to understanding the evolution and maintenance of cleistogamy, these effects have been repeatedly confounded. In an attempt to separate these effects, we compared the performance of progeny derived from the two floral morphs while controlling for the source of pollen. That is, flower type and pollen source effects were assessed by comparing the performance of progeny derived from selfed CH vs. CL and outcrossed CH vs. selfed CH flowers, respectively. The experiment was carried out with the herb *Ruellia nudiflora* under two contrasting light environments. Outcrossed progeny generally performed better than selfed progeny. However, inbreeding depression ranges from low (1%) to moderate (36%), with the greatest value detected under shaded conditions when cumulative fitness was used. Although flower type generally had less of an effect on progeny performance than pollen source did, the progeny derived from selfed CH flowers largely outperformed the progeny from CL flowers, but only under shaded conditions and when cumulative fitness was taken into account. On the other hand, the source of pollen and flower type influenced seed predation, with selfed CH progeny the most heavily attacked by predators. Therefore, the effects of pollen source and flower type are environment-dependant and seed predators may increase the genetic differences between progeny derived from CH and CL flowers. Inbreeding depression alone cannot account for the maintenance of a mixed mating system in *R. nudiflora* and other unidentified mechanisms must thus be involved.

## Introduction

Although plant mating system theory predicts that only either complete selfing or outcrossing can be evolutionarily stable reproductive strategies [1], paradoxically about 42% of flowering plants exhibit a mixed mating system in nature [2]. According to this theory, some plant species will tend to outcross to avoid the negative effects of inbreeding depression on fitness [3], while others will tend toward selfing because it ensures reproduction under conditions adverse to outcrossing [4], as well as the benefit associated with purging deleterious alleles [1]. In either case, inbreeding depression is thought to have a significant effect, with low levels of inbreeding (<50%) promoting selfing and high levels of inbreeding (>50%) promoting outcrossing [5]. The mismatch between theory and empirical evidence regarding the stability of mixed mating systems has recently motivated the search for alternative mechanisms to explain the chronic persistence of mixed mating in plants [6,7]; however, a proper test of these mechanisms is strongly limited by the availability and quality of empirical data [2,7].

Perhaps the most clear-cut example of facilitated mixed mating is dimorphic cleistogamy (hereafter only cleistogamy) [8,9], where a single plant produces both open potentially outcrossed chasmogamous (CH) flowers and also closed, obligately self-pollinated cleistogamous (CL) flowers [10,11]. Typically, CL flowers are energetically less costly, smaller, and produce smaller seeds than CH flowers do [10,11]. Because plants with cleistogamy have specialized flowers for self- and cross-pollination, these plants represent an excellent model for the study of the mechanisms underlying the stability of mixed mating systems. Contrary to expectation, cleistogamous plants have received far less attention than plants with a mixed mating system bearing monomorphic flowers [8]. Also, only a few theoretical models on the stability of mixed mating systems have considered the peculiarities of cleistogamy [2,8]. The most accepted model explaining the stability of cleistogamy suggests that floral dimorphism is maintained because of adaptive phenotypic plasticity; that is, plants maximize fitness by producing less costly and self-pollinated CL flowers under suboptimal conditions, while energetically expensive and potentially outcrossed CH flowers are produced under optimal environmental conditions [12]. Although optimal environmental conditions sometimes coincide with the production of CH flowers (e.g., 13), there is little empirical evidence supporting or refuting adaptive phenotypic plasticity [14]. According to this model, a basic assumption for cleistogamy to evolve is that, at least under certain environmental conditions, progeny derived from CH flowers (hereafter CH progeny) outperforms progeny derived from CL flowers (hereafter CL progeny), with the avoidance of inbreeding depression as the underlying mechanism [8,15]. However, cleistogamous plants generally exhibit low inbreeding depression (ranging from -97% [16] to 43% [17]) and CH flowers are energetically more expensive, but at the same time less fertile than CL flowers [8]. It has been estimated that the fitness advantage of CL progeny is on average 1.21 times that of CH progeny [8]. Therefore, in order to be stable, the forces maintaining CH flower production must be strong enough to counterbalance the advantages of reproduction via obligate selfing (CL) [8]. However, this condition has not been demonstrated in any cleistogamous species studied so far. 

Although the low inbreeding depression reported in the literature for cleistogamous species may be explained by a long history of obligate selfing, indeed most studies have failed to separate the effect of inbreeding *per se* from the effect of flower type i.e., they usually compare performance and fitness between CH and CL progeny assuming that CH flowers are outcrossed (e.g., [18-21], but see 16,17). However, CH flowers in several cleistogamous plant species are self-compatible [15,18,23–25] and therefore the effect of flower type and of the pollen source (selfed vs. outcrossed) is confounded in most studies contrasting the performance or fitness of CH and CL progeny, and this may also bias the estimate of inbreeding depression [26]. Furthermore, the expression of inbreeding depression is usually environment-dependant, with inbreeding effects usually more evident under harsher conditions [27,28]. However, studies looking at the performance of selfed vs. outcrossed progeny (and controlling for flower type) under different environmental conditions are extremely rare for cleistogamous species (see [15,29]).

In this study, we addressed pollen source (self vs. outcrossed) and flower type (CH vs. CL) effects on progeny performance and fitness by conducting appropriate contrasts between floral morphs while controlling for the pollen source. That is, we approached pollen source effects (i.e., inbreeding depression) by comparing progeny from selfed CH vs. outcrossed CH progeny and flower type effects by comparing progeny from selfed CH vs. obligately-selfed CL progeny (CL flowers cannot be outcrossed). We conducted the study under two contrasting light environments (natural light *vs.* a 50% reduction of ambient light) in *Ruellia nudiflora*, a perennial herb with dimorphic cleistogamy. It is known that the light environment affects flowering phenology in this species [13] and it also likely affects the expression of inbreeding depression. In the past few years, some research has shown that herbivores and pathogens preferentially attack selfed progeny [30-33]. However, up to now no study has been conducted on a cleistogamous system. *R. nudiflora* fruit and seeds are attacked before dispersal by the larvae of a noctuid moth [34,35], and the incidence of the attack may be influenced by not only the source of pollen, but also by the flower type because CH and CL fruit and seeds differ morphologically (CH fruit are bigger and have more and heavier seeds than those of CL) [35]. We predict better progeny performance and reduced seed predation in outcrosssed CH progeny relative to selfed CH progeny (pollen source effect). Also, we predict that selfed CH progeny will outperform CL progeny given that CH flowers and seeds receive more resources (CH flowers are far larger and CH seeds much heavier) than those of CL do (flower type effect). Additionally, we expect a stronger effect of the pollen source and flower type on the performance of the progeny in the light-limited environment. 

## Materials and Methods

### Study area and species

The study was conducted in two experimental plots belonging to the Universidad Autónoma de Yucatán (UADY) located in the Xmatkuilt community (20°52’N, 89°36’W, 10 m a.s.l.). The campus is next to a protected natural area where the vegetation is a moderately disturbed tropical dry forest dominated by *Bursera simaruba* (Burseraceae), *Piscidia piscipula* (Fabaceae) and *Lysiloma latisiliquum* (Fabaceae) in the canopy, and *Parmentiera millspaughiana* (Bignoniaceae) in the understory [36]. The climate of the region is warm sub-humid with summer rains; mean annual rainfall and temperature are 850 mm and 26.2 °C, respectively [37].

*Ruellia nudiflora* (Acanthaceae) is a perennial herb with dimorphic cleistogamy (*sensu* Culley and Klooster [10]), meaning that a single plant produces both, CL and CH flowers. CH flowers are larger (2.4±0.3 cm) and CH seeds are heavier (3.42 ± 0.8 mg) than CL flowers (0.4±0.1 cm) and seeds (2.65 ±0.05mg). CL flowers never open and obligately self-pollinate while CH flowers do open and can be outcrossed. Previous hand pollination experiments have shown that fruit set in autonomous selfed (38%), manual cross-pollinated (7%) and open-pollinated CH flowers all differ significantly [38]. CL flowers and fruit are produced during almost the entire reproductive season (April-December) while only one or two brief production peaks (shorter than a month) of CH flowers have been recorded per reproductive season [39]. Pollinators are native and exotic bees (*Apis mellifera*, *Trigona fulviventris*) as well as butterflies (*Microtia elva*). *R. nudiflora*’s lifespan is unknown but is longer than three years. Fruit are dry capsules; CL fruit are typically smaller than CH fruit [34]. Both fruit types are predated by a species of noctuid moth that consumes a large proportion of the seeds in the fruit it attacks [34,35]. Pollinators and seed predators move freely between the experimental area and surrounding vegetation. *R. nudiflora* is a light-demanding weed and therefore occurs preferentially in open and disturbed spaces such as roadsides, agricultural fields, urban areas and disturbed forest [13,40]. *R. nudiflora* occurs naturally in the study area, where it is often visited by pollinators and attacked by seed predators. 

 The study species is highly abundant at the site and not under any special protection category according to national or international law. The experimental plots are not part of the protected area, thus, no permit was required from the local government. 

### Experimental design

In early March 2011, 20 adult *R. nudiflora* plants were transplanted from the Molas municipality to a greenhouse in the Universidad Autónoma de Yucatán (20 km away). The vegetation in Molas is a disturbed forest where *R. nudiflora* occurs naturally. Only apparently healthy plants of similar height and growing at least 2 m apart from each other were collected. The vegetation, climate and associated insect fauna (pollinators and predators) are very similar in the area of plant origin and the area where the study was conducted [13]. Once in the greenhouse, plants were planted in 1.5 L plastic pots with substrate from the site of origin and watered as needed. In April and May 2011 most of the plants started producing CH flowers and we conducted two hand-pollination treatments: outcrossing (hereafter CH-C) and selfing (hereafter CH-S). For CH-C, the source of pollen was a flower from a different plant in the greenhouse. For CH-S, pollen from the same flower was used. Hand pollinations were conducted shortly after pollen release (0800-0900 h), and pollen grains were gently placed on the stigma until it was apparently saturated. To avoid pollen contamination we removed the corolla and stamens of hand-pollinated flowers before (0730-0830 h) or just after pollen release (0800-0900 h) for CH-C and CH-S treatments, respectively. We did not see any insects visiting the flowers of the experimental plants inside the greenhouse. Treated flowers were labeled and fruit collected and stored in individual bags when ripe under laboratory conditions. Simultaneously, ripe CL fruit were collected and stored together with CH-C and CH-S fruit. CH and CL fruit can be easily told apart because the former usually bears remnants of the style and are bigger than CL fruit [13,35]. As some plants did not produce CH flowers or set any fruit, only nine maternal families (i.e., a group of CH-C, CH-S & CL seeds obtained from a given maternal plant) were completed. In July 2011 from 10 to 26 seeds per pollen source (i.e., CH-C, CH-S, CL) per maternal family were sown in conditions similar to those of their maternal plants. Seed germination did not differ statistically among pollen sources (generalized linear model with a binomial error distribution; χ^2^_2_=0.25, P=0.88). As seeds were germinated under greenhouse conditions, germination was not used as a measure of performance or fitness in posterior analyses.

In late October 2011, seedlings with at least one pair of fully expanded true leaves were transplanted to the experimental plots (6 x 8 m). 343 plants were planted randomly in the experimental plots, separated by 15 to 25 cm. Two plots (2 m apart) were needed to accommodate all the plants: 180 in the first plot and 163 plants in the second. Approximately half of each plot was shaded with light-neutral nylon mesh which reduces ambient light by 50%. Thus, 99 (30 CH-C, 35 CH-S, 34 CL) and 83 (29 CH-C, 25 CH-S, 29 CL) plants were shaded in plot one and plot two respectively, while 81 (22 CH-C, 31 CH-S, 28 CL) and 80 (26 CH-C, 28 CH-S, 26 CL) plants received ambient light (hereafter referred to as open) in plot one and plot two. The mean number of plants per maternal family was 38 (range 23-52) at the beginning of the experiment. Plants were watered evenly as needed during the experiment. The number of CH flowers and ripe fruit produced per plant and the height of each plant were recorded once a week. CL flowers were not recorded because it was not possible to differentiate CL flowers from early CH floral buds. Ripe fruit were collected, weighed and dissected under the microscope in search of evidence of predation (exit holes, predator parasitoids, cocoons or excrement), 98% of the fruit examined (n=642 fruit) were CL fruit. In late July 2012, after 9 months of monitoring, 271 plants survived. The aerial part of 260 plants was harvested, dried and weighed in order to estimate the aboveground biomass. The other 11 surviving plants were not weighed because we lost some of the plant material during transportation and the label went missing from other plants during the experiment.

### Statistical analyses

The effects of the pollination treatment (three levels: CH-C, CH-S and CL), light availability (two levels: shade and open), maternal family (nine maternal families) and second order interactions on the total number of CH flowers and fruit produced during the experiment, as well as on individual survivorship at the end of the study (a binary variable: live vs. dead), were assessed with generalized linear mixed-effects models (three different models in total). As we were interested in assessing variation among maternal families as well as how consistent the effects of pollen source and light availability were on different families, this factor was considered fixed instead of random (see [41] for a description of fixed vs. random effects). In all models, the shade/open sub-plot nested in the main plot (one or two) was included in the random part of the model. A Poisson and binomial error distribution was assumed for the number of reproductive structures (number of flowers and fruit) and plant survivorship, respectively. All these models were fitted with the penalized quasi-likelihood method [42]. 

The effects of the three main factors (pollination treatment, light availability and maternal family) on the proportion of fruit predated per plant were also assessed. For this response variable, only the pollination treatment x light interaction was tested because some plants did not set any fruit leading to an incomplete design. The effects of the three main factors mentioned and their second order interactions on aboveground biomass and height were assessed with linear mixed-effects models. The random part of this model was as for the previously described models. Linear mixed-effects models were also used to fit another model where the mean fruit weight per plant was the response variable, the same explanatory variables and interaction were used for the proportion of fruit predated. Linear mixed-effects models were fitted with the restricted maximum likelihood method [41]. Following the suggestion of Crawley [41], we visually examined the distribution of residuals to assess model fit, which we found satisfactory in all models. All statistical analyses were run in R 2.14.0 software [43]. When a statistically significant difference among levels of pollen source was found, a posteriori contrasts were performed to identify differences between pair of means [41]. The CH-C vs. CH-S contrast tells us about the effect of the source of pollen, while the CH-S vs. CL contrast tells us about the effect of flower type [16]. The CH-C vs. CL contrast is not of interest because the effects of pollen source and flower type are confounded. 

 The inbreeding depression index was calculated as follows: Inbreeding depression index= 1- (W CH-S / W CH-C), where W CH-S and W CH-C are the fitness of CH-S and CH-C progeny, respectively [16]. Three different measures of fitness were considered [16]: aboveground biomass, survivorship and a cumulative fitness (survivorship x number of fruit). Positive values indicate inbreeding depression while negative values indicate outbreeding depression. In an analogous way, the relative effects of flower type, i.e., the extent to which CH-S progeny outperformed (positive values) or underperformed (negative values) CL progeny, were calculated as follows: Flower type effect index= 1- (W CL / W CH-S), where W CL is the fitness of CL-progeny and W CH-S is as previously described [16]. Also, to assess flower type effects the three fitness measures mentioned above were considered in the analysis.

 Raw data are available upon request from the author for correspondence. 

## Results

The pollination treatment affected progeny flower production, aboveground biomass, plant height, fruit weight and fruit predation (Table 1). In contrast, no significant difference was found in fruit production or survivorship (Table 1). Mean values of aboveground biomass, plant height and fruit weight were significantly greater in CH-C than in CH-S and CL progeny (Table 2), suggesting a strong pollen source effect on the performance of progeny (i.e., outcrossed progeny outperformed selfed progeny). Also, CH-S and CL progeny did not differ in any of these three variables, which implies there was no significant flower type effect (Table 2). However, CH-S progeny produced more flowers and were attacked to a greater extent by predators than CH-C and CL progeny, suggesting significant pollen source and flower type effects on these variables (i.e., the greatest flower production and predator attack occurred in selfed CH progeny; Table 2). CH-C and CL progeny did not differ for these two variables either (Table 2).

**Table 1:** Results of the statistical analyses.

	**Response variable**						
**Source of variation**	**Flowers**	**Fruit**	**Dry weight**	**Height**	**F. weight**	**Predation**	**Survivorship**
**Pollination**	*F*_2,232_ = 3.9*	*F*_2,232_ = 2.9	*F*_2,220_ = 5.7**	*F*_2,232_ = 5.4**	*F*_2,168_ = 6**	*F*_2,168_ = 8.7**	*F*_2,303_ = 1.1
**Light**	*F*_1,1_ = 217**	*F*_1,1_ = 6.7	*F*_1,1_ = 15.8	*F*_1,1_ = 0.1	*F*_1,1_ = 0.9	*F*_1,1_ = 1.8	*F*_1,1_ = 0.1
**Family**	*F*_8,232_ = 5.8**	*F*_8,232_ = 4.6**	*F*_8,220_ = 3.5**	*F*_8,232_ = 1.9	*F*_8,168_ = 1.2	*F*_8,168_ = 1.1	*F*_8,303_ = 0.9
**Pollination x light**	*F*_2,232_ = 1.3	*F*_2,232_ = 0.9	*F*_2,220_ = 0.7	*F*_2,232_ = 0.7	*F*_2,168_ = 0.5	*F*_2,168_ = 2.6	*F*_2,303_ = 0.8
**Pollination x family**	*F*_16,232_ = 1.7	*F*_16,232_ = 1.4	*F*_16,220_ = 1.2	*F*_16,232_ = 1.2	*-*	-	*F*_16,303_ = 1.3
**Light x family**	*F*_8,232_ = 3.9**	*F*_8,232_ = 2.0	*F*_8,220_ = 1.1	*F*_8,232_ = 1.4	*-*	-	*F*_8,303_ = 0.9

Mixed-effects models (linear and generalized linear) were used to assess the effect of pollination treatment (Pollination, three levels: chasmogamous cross-pollinated, chasmogamous self-pollinated and cleistogamous self-pollinated) and light availability (Light, two levels: ambient light and 50% of ambient light) on flower number (Flower), fruit number (Fruit), aboveground biomass (Dry weight), plant height (Height), fruit weight (F. weight), fruit predation (Predation) and plant survival (Survivorship) in *Ruellia nudiflora*. Hand pollination was performed for nine maternal families (Family). For fruit weight and predation, pollination x family and light x family interactions could not be tested because some plants did not produce any fruit.

*P<0.05, **P<0.01

**Table 2:** Progeny performance and predation (Trait) given different flower types and pollen sources (Pollination treatment).

	**Pollination treatment**		
**Trait**	**CH-C**	**CH-S**	**CL**
Flowers (number)	15.9**^a^** (1.7)	17.8**^b^** (1.9)	14**^a^** (1.6)
Fruit (number)	1.6**^a^** (0.2)	1.8**^a^** (0.3)	1.6**^a^** (0.2)
Dry weight (g)	11.9**^a^** (0.8)	9.2**^b^** (0.7)	8.9**^b^** (0.6)
Height (cm)	28.1**^a^** (1.3)	25.1**^b^** (1.2)	23.1**^b^** (1.1)
Fruit weight (mg)	15**^a^** (0.5)	13.7**^b^** (0.5)	12.4**^b^** (0.5)
Predation (proportion)	0.4**^a^** (0.04)	0.6**^b^** (0.05)	0.4**^a^** (0.05)
Survivorship (%)	85**^a^**	72**^a^**	80**^a^**

Mean (SE) number of flowers (Flowers), number of fruit (Fruit), dry weight of the aboveground part of the plant (Dry weight), the height of the plant (Height), fruit weight (Fruit weight), proportion of fruit predated (Predation) and percent survivorship of plants produced by different fruit types (chasmogamous [CH] or cleistogamous [CL]) and sources of pollen (outcrossed [C] or self-pollinated [S]). Different superscript letters indicate statistically significant differences. For survivorship, a binary response variable (live or dead) was used as the response variable, so SE was not calculated.

Light significantly affected the number of flowers (Table 1) with mean values greater for plants in the open than those under the shade cloth (Table 3). The number of fruit, aboveground biomass, plant height, fruit weight, fruit predation and survivorship did not differ between light environments (Table 1 & 2).

**Table 3:** Progeny performance and fruit predation (Traits) under two contrasting levels of light availability (Light).

	**Light**	
**Trait**	**Open**	**Shade**
Flowers (number)**	27.1 (1.5)	5.7 (0.5)
Fruit (number)	2.7 (0.3)	0.7 (0.1)
Dry weight (g)	11.5 (0.7)	8.6 (0.5)
Height (cm)	25.1 (0.9)	25.6 (0.9)
Fruit weight (mg)	14.4 (0.3)	12.2 (0.6)
Predation (proportion)	0.5 (0.04)	0.4 (0.05)
Survivorship (%)	80	78

Mean (SE) number of flowers (Flowers), number of fruit (Fruit), dry weight of aboveground part of plant (Dry weight), height of the plant (Height), fruit weight (Fruit weight), proportion of fruit (Predation) and percent survivorship of plants grown under contrasting light conditions: ambient light (Open) and 50% ambient light (Shade). For survivorship, a binary response variable (live or dead) was used as the response variable, so SE was not calculated.

** *P*<0.01

No statistical difference among maternal families was found in terms of plant height, mean fruit weight, fruit predation, or survivorship (Table 1). The number of flowers and fruit as well as aboveground biomass differed among some maternal families (Table 1, see also Figure S1). No interaction was statistically significant except light x maternal family for flower production (Table 1). However, despite the variation in the magnitude of the difference, in every maternal family the effect of light on flower production was the same: flower production was greater in the open than under the shade cloth (see Figure S2). 

In the open, inbreeding depression was very low for survivorship (8%) and cumulative fitness (1%), but moderate for aboveground biomass (25%; Table 4). With the exception of aboveground biomass, inbreeding depression was much higher under the shade cloth (Table 4), where inbreeding depression was similar and moderate (21 and 23%) for survivorship and aboveground biomass while the greatest value (36%) was for cumulative fitness (Table 4). In the open, the flower type effect indexes were negative (meaning CL-derived progeny had a fitness advantage) but in general very low (1-7%) for the three fitness measures: aboveground biomass, survivorship and cumulative fitness (Table 4). Under the shade cloth, the flower type index was negative for survivorship and slightly positive (0.09) for aboveground biomass. As occurred with the effect of inbreeding depression, the effect of flower type was most notable in the measure of cumulative fitness (Table 4). 

**Table 4:** Inbreeding depression and flower type effect indexes in two contrasting light environments: open and under shade cloth that reduces ambient light by 50% (shade).

	**Inbreeding depression**		**Flower type effect**	
**Fitness measure**	**Open**	**Shade**	**Open**	**Shade**
Dry weight	0.25	0.23	-0.01	0.09
Survivorship	0.08	0.21	-0.07	-0.16
Cumulative fitness	0.01	0.36	-0.05	0.14

Three measures of fitness were considered: above ground dry weight (Dry weight), survival probability (Survivorship) and cumulative fitness (Survivorship x Fruit number).

## Discussion

In cleistogamous plants, the relative effect of pollen source (selfed vs. outcrossed) and floral dimorphism (CH vs. CL) is naturally confounded because most of cleistogamous species are self-compatible [15,16,29]. Despite the fact that inbreeding depression and floral dimorphism *per se* have been identified as key aspects for understanding the evolutionary stability of dimorphic cleistogamy [8], most of the previous studies have failed to separate these effects [18-22]. In this study we have shown clearly that pollen source has a stronger relative effect (compare CH-C *vs.* CH-S in Table 2) than flower type (compare CH-S vs. CL in Table 2) on progeny performance in *Ruellia nudiflora*. In general, outcrossed progeny outperformed selfed progeny and, as predicted, this effect was more evident under suboptimal, shaded conditions. However, inbreeding depression as estimated in this study is generally below 50% (1-36%) and therefore, inbreeding depression alone cannot account for the maintenance of costly CH flowers in *R nudiflora*. 

### Pollen source effects

In cleistogamous plants, reproduction via CL seeds has several advantages including reproductive reliability (i.e., the ability to set seed under adverse environmental conditions), reduced energetic cost, greater fertility and the purging of deleterious alleles (but Muller's ratchet and mutational meltdown are also possible scenarios). Therefore, the maintenance of costly CH flowers remains one of the great unanswered questions in Evolutionary Biology [2,8]. Theoretical models of the evolution of selfing predicts that inbreeding depression must be as high as or greater than 50% to prevent selfing from evolving to fixation [1,44]. Inbreeding depression as detected in this study is in general lower than 50% (see Table 4), even in stressful shaded environments. However, the level of inbreeding depression seen in *R. nudiflora* is also among of the highest reported for a cleistogamous plant. Previous studies on cleistogamous species estimated that inbreeding depression ranges from -97% (*Viola canadensis* [16]) to 43% (*V. septembola* [17]), when accounting for flower type. Inbreeding depression seen in *R. nudiflora* could not alone counterbalance the trend to selfing fixation due to the reproductive advantages of CL in this plant species; therefore, other aspects not evaluated in this or other studies may play a complementary role for the maintenance of CH flowers.

Shading represents a suboptimal condition for *R. nudiflora* as suggested by the strong and negative effect of shade on flower production (see Table 3). While the effect of shading did not interact with pollination treatment (see Table 1), some interesting patterns emerged when pollination treatment was broken down into pollen source and flower type effects (see Table 4). Under shaded conditions, inbreeding depression for cumulative fitness increased 35% relative to plants in the open. This finding agrees with the view that inbreeding depression increases under harsher conditions [27,28]. Only a couple of previous studies have properly addressed inbreeding depression in different environments for cleistogamous plants, and the few studies available have reported contradictory results [17,29]. Inbreeding depression only varied slightly (∆ 3%) in *V. pumilla* or went from negative to slightly positive (14%) in *V. stagnina* under harsher (sterilized soil) vs. more benign (unsterilized soil) conditions [29]. *Viola septembola* exhibited the opposite pattern to that seen in *R. nudiflora*, with greater inbreeding depression (40%) under benign (i.e., greenhouse) compared to (25%) the more stressful conditions in the field [17]. Given that studies are very scarce so far, and the fact that all of the previous studies have been conducted on *Viola* species, no pattern can be depicted at present regarding inbreeding depression or the influence of the environment on it, in cleistogamous species.

### Flower type effects

Considering that CH-S and CL progeny did not differ in hardly any of the performance indicators used in this study (see Table 2) and the fact that most of the flower type effect indexes were only slightly different from zero (see Table 4), we suggest that the effect of flower type on the performance and fitness of progeny is only minor in *R. nudiflora*. A noteworthy exception was the effect of flower type on cumulative fitness (14%), but only under shade cloth. This result may suggest that, as for the pollen source effect, the effect of flower type is reinforced under shaded conditions but only in a reduced number of variables. Therefore, the effects of pollen source and flower type are context-dependant in *R. nudiflora*.

### Seed predation and maternal family effect

Another exciting result was the greater seed predation seen in CH-S progeny relative to CH-C and CL. This result suggests that not only pollen source but also flower type affects fruit predation. The results suggest that CH-S fruit are attacked more frequently by predators. Previous studies have shown that inbreeding negatively affects resistance to herbivory [30-33] and this seems to be the case for *R. nudiflora*. However, this mechanism alone does not explain why predators preferred CH-S over CL progeny. One possibility is that, because more resources are allocated to CH flowers and derived seeds [8], CH progeny might produce fruit of greater quality (i.e., with more and/or larger seeds and with more nutrients) for predators than CL progeny. An important implication of this result is that seed predators might increase the genetic advantage of CH progeny over CL by selecting against CH-S progeny. 

 Maternal family significantly affected three of seven performance measures, however, even in these variables families showed little variation (Figure S1). Also, maternal family did not interact with the pollination treatment (Table 1), suggesting that the relative effects of pollen source and flower type are maintained across different families in nearly all of the performance measures. Therefore, maternal family appears to be of little importance with respect to the relative effects of pollen source and flower type on the performance and fitness of progeny as well as seed predation.

## Conclusions

There is a significant advantage in terms of the performance of outcrossed over selfed progeny. The latter is also more likely to be attacked by predators. Inbreeding depression in *R. nudiflora* ranges from low to moderate (1-36%); therefore, this mechanism by itself cannot account for the maintenance of CH flowers in this species. Predispersal seed predation by filtering selfed CH progeny may increase genetic differences between CH and CL progeny. Flower type also influences seed predation and performance under certain conditions (shade). However, the importance of flower type relative to the effect of pollen source is minor. Pollen source and flower type effects are context-dependant; the effects are stronger under suboptimal shaded conditions. Future research—in addition to separating pollen source and flower type effects—should evaluate plant performance and fitness in different environmental contexts. It may be worth re-evaluating inbreeding depression using lifespan fitness. It would also be interesting to look at inbreeding depression experimentally, with seed predators excluded. Other mechanisms besides inbreeding depression may be maintaining CH flowers in cleistogamous plants, and these mechanisms should be identified in future research.

## Supporting Information

Figure S1 **Flower, fruit and biomass production per maternal family.** Bars show mean (± 1 SE) flower number (A), fruit number (B) and dry weight in grams of the aboveground part of the plant (C) observed in 9 maternal families of the weed *R. nudiflora*.
(TIF)

Figure S2 **Interaction between light and maternal family in flower production.** Symbols show mean CH flower production (± 1 SE) per plant for 9 different maternal families of the weed *R. nudiflora* under two levels of light availability: ambient light (open) and 50% of ambient light (shade).
(TIF)
