# Supplementary figures and images for: The Effect of Pollen Source vs. Flower Type on Progeny Performance and Seed Predation under Contrasting Light Environments in a Cleistogamous Herb
Source: PLoS One. 2013 Nov 15;8(11):e80934. doi: 10.1371/journal.pone.0080934 (PMC3829907; doi:10.1371/journal.pone.0080934)

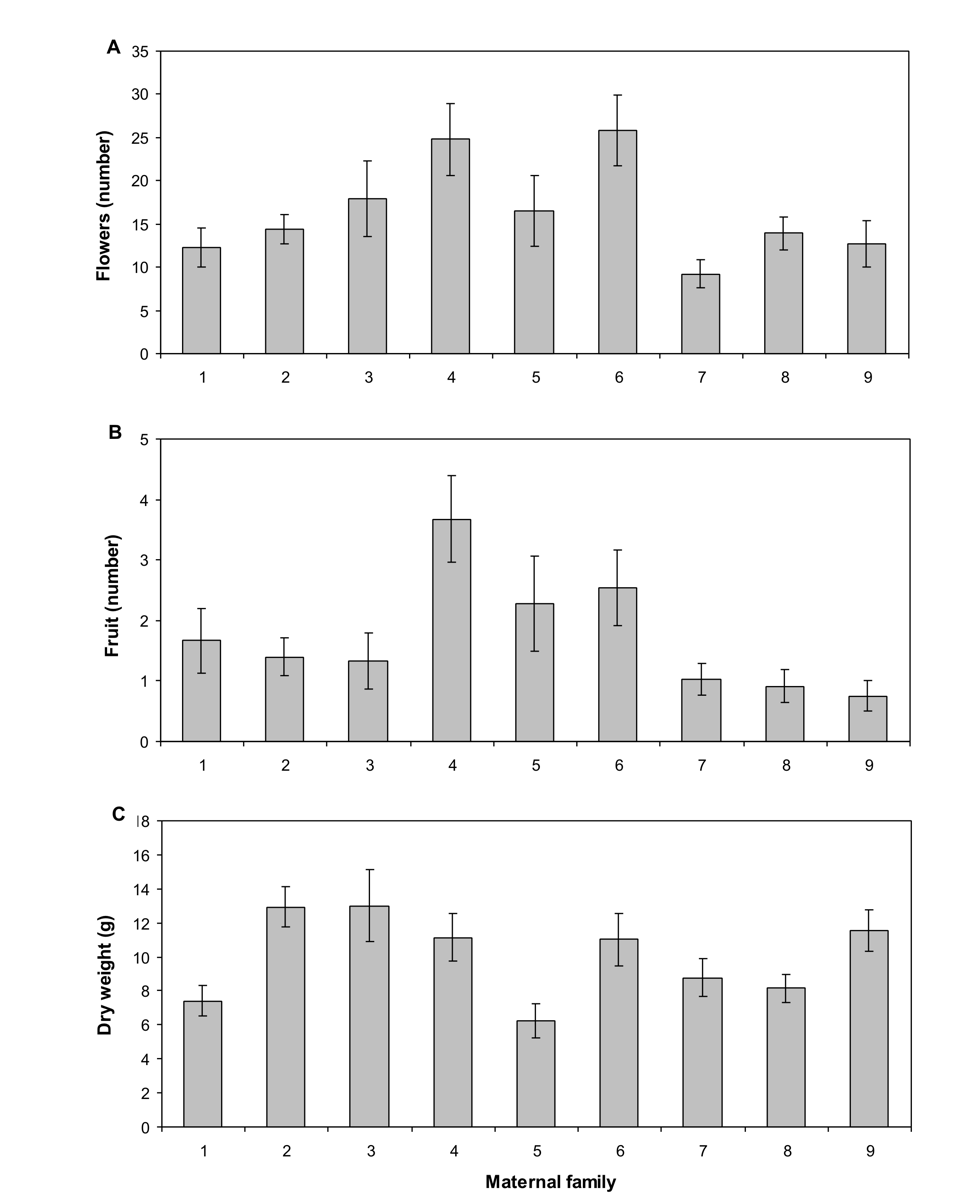

Supplement: Figure S1 — Flower, fruit and biomass production per maternal family. Bars show mean (± 1 SE) flower number (A), fruit number (B) and dry weight in grams of the aboveground part of the plant (C) observed in 9 maternal families of the weed R. nudiflora. (TIF) [file pone.0080934.s001.tif]

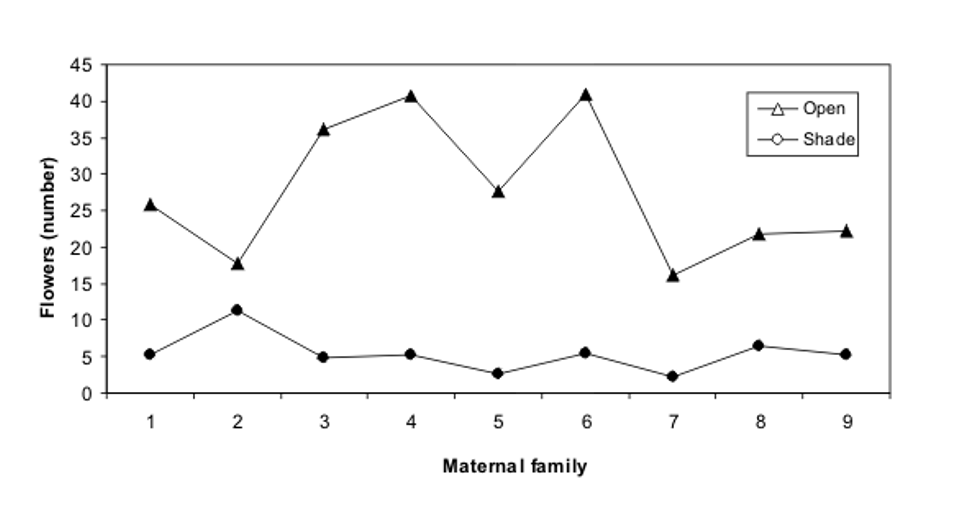

Supplement: Figure S2 — Interaction between light and maternal family in flower production. Symbols show mean CH flower production (± 1 SE) per plant for 9 different maternal families of the weed R. nudiflora under two levels of light availability: ambient light (open) and 50% of ambient light (shade). (TIF) [file pone.0080934.s002.tif]
